# Smoking and the risk of prostate cancer: a review of risk and disease progression

**DOI:** 10.1186/s41021-025-00338-8

**Published:** 2025-10-09

**Authors:** Ishvaria Sundaresan, Nallasivam Palanisamy, Radha Saraswathy

**Affiliations:** 1 Department of Biomedical Sciences, School of Biosciences & Technology, Vellore Institute of Technology, Vellore, Tamil Nadu India; 2 Department of Urology, Henry Ford Health, Detroit, Michigan USA

**Keywords:** Prostate cancer, Smoking, Risk factors, Hormonal imbalance, Smoking cessation, Public health

## Abstract

**Background:**

Prostate cancer is still the most common malignancy affecting men worldwide, with causes ranging from genetics to environmental and lifestyle factors. This review narrows its attention to investigate smoking as a risk factor for the start and progression of prostate cancer. While age, ethnic differences, family history, and genetic abnormalities such as BRCA1 and BRCA2 remain important, smoking—particularly long-term and heavy use—emerges as a modifiable risk factor that needs deeper consideration. Though this review attempts to offer a worldwide perspective on smoking and prostate cancer risk, we also include a focus on new research from India, given the country’s particular regional patterns and growing evidence.

**Methods:**

A systematic review of PubMed, Scopus, and Web of Science was undertaken using "smoking" and "prostate cancer." The criteria for selecting articles were relevancy, developing research, and accessibility. The exclusion criteria eliminated non-human research and associated issues. This study examined worldwide patterns, however primarily focused on tobacco use and prostate cancer in India according to recent findings. Regional research emphasised smoking and prostate cancer risk patterns and mechanisms.

**Results:**

Tobacco use is still a substantial risk factor for several malignancies, including prostate cancer. Globally, smoking has been associated with an increased risk of getting prostate cancer, with research indicating that smokers had a greater prevalence of aggressive illness. Tobacco usage is very common in India owing to a variety of cultural, societal, and economic variables; hence it is a key focus of this research. The effect of tobacco in prostate cancer risk in India is still being studied, and the data shows that smoking in the Indian population may worsen the development and progression of prostate cancer, similar to worldwide patterns but with regional differences.

**Conclusions:**

Understanding how smoking affects prostate cancer may improve prevention and early diagnosis, which has public health consequences. These methods may involve targeted screening or lifestyle changes. This review emphasis smoking as a key prostate cancer risk factor, focusing on new Indian findings. More research is required to assess smoking's full impact on prostate cancer risk, particularly in different populations and locations.

**Supplementary Information:**

The online version contains supplementary material available at 10.1186/s41021-025-00338-8.

## Introduction

Tobacco smoking is known to cause various cancers, including lung and bladder cancer [1]. However, its relationship with prostate cancer (PCa) is unclear. While many studies have not identified a direct connection between smoking and the onset of the disease, there is emerging evidence suggesting that smokers might be at a higher risk of fatal PCa [2]. Interestingly, research has noted that smoking may have a protective effect against localized PCa, but does not show a significant association with advanced stages of the disease [3–5].

**Fig. 1:**
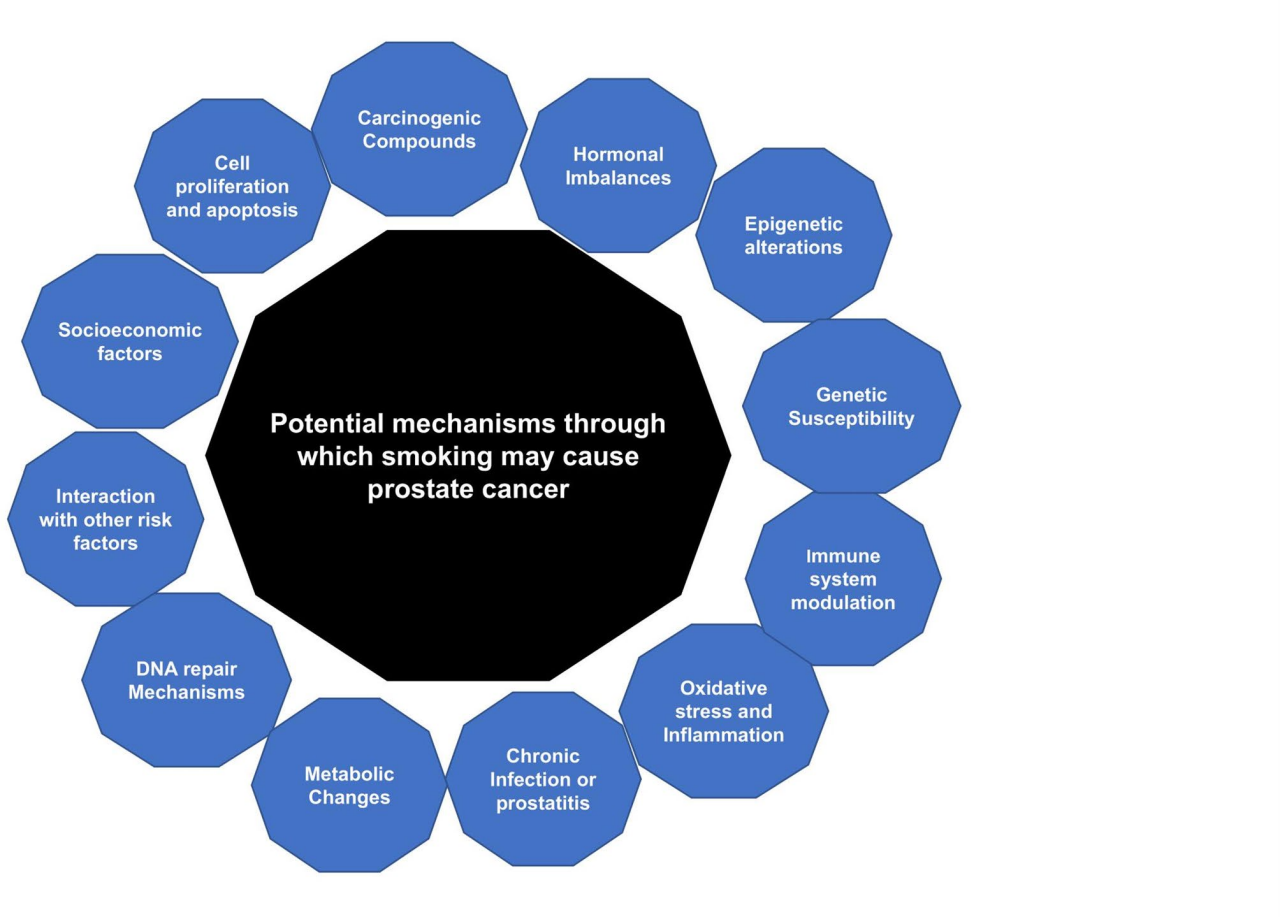
Potential mechanisms through which smoking may contribute to the development of prostate cancer. The exact mechanism by which smoking influences prostate cancer risk are still being researched, and multiple factors likely interact. Understanding these complexities is an ongoing area of research

Recent research suggests that cigarette smoking not only increases the incidence of high-grade PCa and prostate cancer-specific mortality, but it also hinders early detection attempts. An analysis of the 2018 Behavioural Risk Factor Surveillance System found that current smokers are substantially less likely to obtain PSA screening as recommended than former or never smokers. Reduced screening rates may delay PCa detection, lowering outcomes for smokers [6]. These results highlight a complex interaction between behavioral and biological factors, emphasizing the need for further study on tobacco components’ biological interactions with prostate cells and tissues. Smoking may accelerate disease progression through various biological mechanisms and may also compromise treatment efficacy [7]. The potential mechanisms of smoking that may cause PCa development are illustrated in Fig. 1. These potential factors show the extensive harm smoking can cause, emphasizing the health risks and far-reaching consequences of smoking. Numerous population-based cohort and patient studies have associated smoking with increased PCa progression and mortality [8–14]. However, patient studies that only analyze cases may be biased if the exposure is considered a disease risk. Population-based research seems to be bias-free, indicating that holistically evaluating cohorts and patients may better understand smoking and PCa mortality. Potential mechanisms through which smoking may contribute to the development of prostate cancer. The exact mechanism by which smoking influences prostate cancer risk are still being researched, and multiple factors likely interact. Understanding these complexities is an ongoing area of research.

### Comparative global and regional patterns of smoking prevalence

Smoking is a major global public health challenge. According to the World Health Organization, as of 2024, there are approximately 1.25 billion adult tobacco users globally, with the number expected to rise to about 1.3 billion by 2025. Population growth continues to sustain this high tobacco burden worldwide [15;16]. Despite a significant decline in smoking rates from 27% in 2000 to 19% in 2022, major regional differences remain. The Western Pacific and European regions have the highest adult smoking rates—about 27% and 25%, respectively—while Africa and the Eastern Mediterranean have the lowest rates, at 9.5% and 4% [15;17]. Anti-smoking measures aimed at adolescents have helped to reduce smoking in younger age groups, but prevalence remains high among older adults who started smoking before becoming aware of its health concerns. In India, according to the Global Adult Tobacco Survey the tobacco consumption is particularly widespread, with nearly 267 million adults aged 15 and above or 29% of the adult population, consuming different forms, including both smoked and smokeless products. Prevalence among men was 42.4% and among women 14.2% [18]. The predominance of bidis, chewable tobacco, and illicit cigarettes further complicates tobacco control efforts in India. These global and regional smoking patterns are critical to understanding prostate cancer risk, as smoking—especially long-term and heavy use—is increasingly recognized as a modifiable risk factor influencing the initiation and progression of prostate cancer. This underscores the need to contextualize smoking prevalence within regional and demographic frameworks to accurately assess its impact on the prostate cancer burden globally and in India.

### Regional trends and emerging evidence on smoking-associated prostate cancer in India

India’s remarkable regional diversity is reflected in the epidemiology of prostate cancer, with significant variations in incidence and risk factors across its cities and states. Recent data from the National Cancer Registry Programme (NCRP) demonstrate that urban centers—including Delhi, Mumbai, Bangalore, and southern districts like Kollam and Thiruvananthapuram—report substantially higher age-adjusted incidence rates (AAR) compared to rural areas [19]. This urban predominance is closely linked to lifestyle factors, particularly tobacco use and smoking, which are more prevalent in metropolitan environments [20, 21]. While early Indian studies did not consistently find a significant association between smoking and prostate cancer [22], recent research and meta-analyses now confirm that smoking is a significant modifiable risk factor, especially with longer duration and greater intensity of use [23, 24]. These findings highlight the importance of targeted health interventions in Indian cities, particularly focusing on smoking cessation and lifestyle modifications, to address the rising incidence of prostate cancer (Table 1).

**Table 1:** Regional trends specific to India

S.No	Author	Study Period	Study Region(s)	Key Finding	Smoking as Risk Factor
1	Cai et al., (2025) [25]	1990–2021	Global & Regional, includes India	Smoking increases prostate cancer mortality risk; urban areas with higher burden; risk linked to duration and intensity of smoking.	Yes
2	Sankarapillai et al., (2024) [26]	2012–2019	Delhi, Mumbai, Bangalore, Kollam, Thiruvananthapuram, Kamrup Urban (Urban centers in India)	Significant rise in prostate cancer incidence in urban centers; linked .to lifestyle including smoking	Implicit (linked to lifestyle)
3.	Kulothungan et al., (2024)[27]	Not stated	India-wide (meta-analysis)	Meta-analysis confirms tobacco use significantly increases prostate cancer risk among Indian populations	Yes
4.	Sharma et al., (2023)[28]	Not stated	Jammu region, J&K	Positive association between duration of smoking and prostate cancer risk; longer duration correlated with higher risk.	Yes

### Prostate cancer incidence worldwide

Prostate cancer is the second most frequent cancer in men and the sixth leading cause of cancer deaths worldwide, with an estimated 1.4 million new cases in 2020 [29]. North America and Western Europe have the highest rates, which can be attributed to improved screening methods such as the PSA test and more awareness. Asian countries have lower incidence rates, which could be related to diet, genetics, or a lack of screening [30]. In parts of Africa, data is limited. Still, there is evidence of significant variation, with some areas reporting increasing incidence: Latin America and the Caribbean have modest rates with country-specific variances [31]. Age is a significant risk factor because it typically affects males over 65 and worsens with age. Regardless of geographical location, ageing populations, lifestyle changes, and improved detection are all contributing to an increase in prostate cancer prevalence worldwide [32]. Widespread PSA testing affects incidence rates and geographical disparities. Global prostate cancer incidence is complicated, requiring continuing study and context-specific treatment [33]. Genetic, environmental, Lifestyle, and healthcare variables are involved. Global prostate cancer diagnoses are expected to reach 2.3 million by 2040, with 740,000 deaths [34]. Prostate cancer prevalence increased 33.5% in 2020 in Europe, followed by Asia (26.2%) and Northern America (16.9%). Africa (6.6%) and Oceania (1.6%) witnessed much lower rates [29]. The average rate was 36.0 per 100,000 men. Latin America, Northern America, the Caribbean, Europe, and Oceania all had age-standardized incidence rates (ASIRs) higher than 59 per 100,000 men. However, in Africa and Asia, ASIRs were less than 30 per 100,000 men. The age-standardized death rate shifted geographical biases. Africa dominated in ASMR, followed by Latin America, the Caribbean, Europe, Oceania, North America, and Asia. Prostate cancer incidence varies by ethnicity and geographic region [35].

### Global prostate cancer burden attributable to smoking by age

**Fig. 2:**
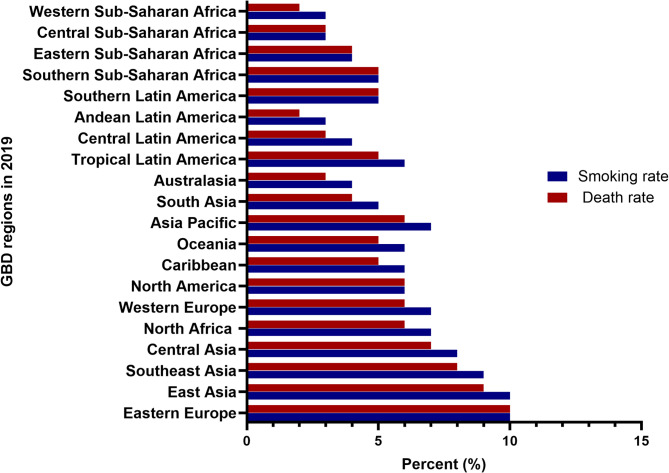
The figure depicts the percentage rate of smoking and prostate cancer deaths in 21 Global Burden of Disease Study (GBD) regions in 2019. The bars in the graph represent the percentage of prostate cancer deaths attributable to smoking rates in each region. The x-axis displays the different 21 GBD regions, while the y-axis shows the percentage of prostate cancer deaths attributable to smoking rates

**Fig. 3:**
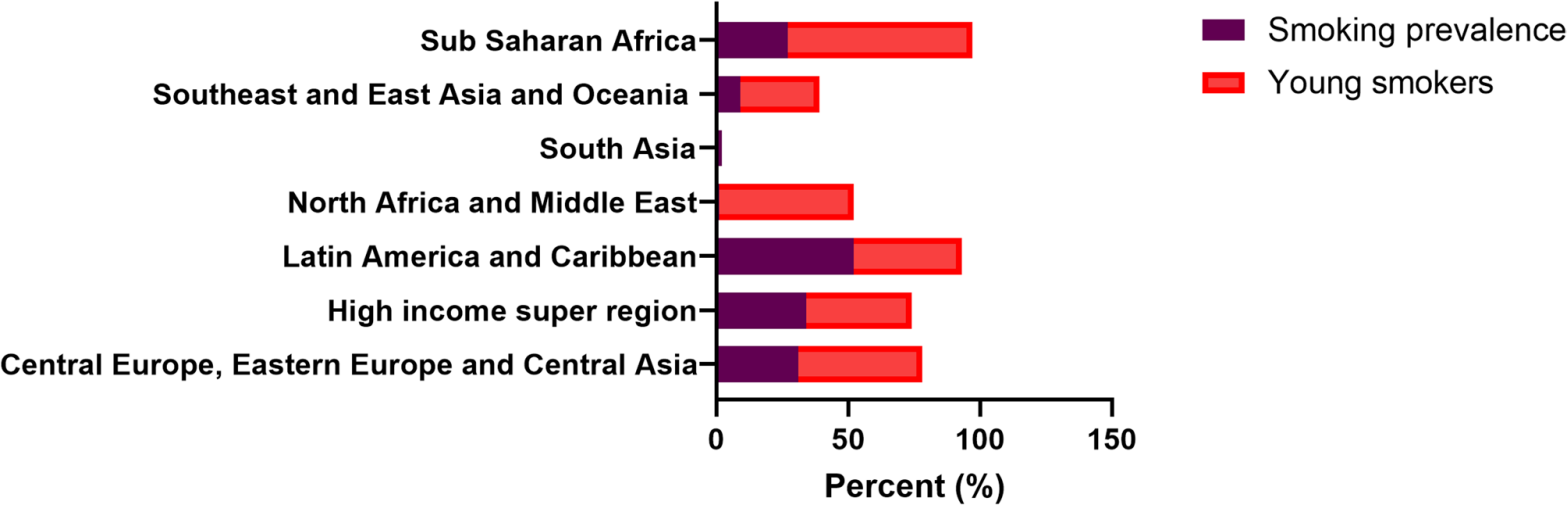
Smoking Prevalence among Youth in Africa, Asia, America, the Caribbean, and Europe. This figure shows the proportion of youth smokers in different regions worldwide. The bars represent the percentage of youth smokers. The highest smoking prevalence among youth is observed in Latin America and the Caribbean (52.1%), followed by Europe (30.7%), Sub-Saharan Africa (26.8%), Asia and Oceania (8.9%), South Asia (2.1%), and the least recorded in North Africa (1.5%)

Although smoking is a well-known risk factor for a variety of illnesses, its influence on prostate cancer incidence remains unknown. The 2019 worldwide Burden of Disease (GBD) research, which examined 369 illnesses and 87 risk factors from over 200 nations, is the first to thoroughly evaluate smoking’s impact on the worldwide prostate cancer burden. This study adds information on how smoking affects prostate cancer rates and highlights the need for focused cancer prevention and control measures [36] It explores smoking’s significant contribution to prostate cancer burden and its global distribution (Fig. 2). In 2019, the number of deaths from prostate cancer linked to smoking followed a pattern: it initially rose, then declined with age. Most deaths occurred in the age range of 70–84, peaking between 75 and 79, with higher numbers in regions with higher socio-demographic index (SDI). Between the ages of 40 and 95, the death rate progressively increased. Globally, However, the death rate fell across all age categories in 2019, with the 75–79 age group seeing the greatest fall. The inclusion of smoking prevalence in younger age groups (15–24) has no apparent relationship to prostate cancer risk. Still, it does help to illustrate worldwide smoking patterns and their possible long-term consequences. Furthermore, an estimated 155 million people aged 15 to 24 smoked in 2019, with a 20.1% rate among men. In 120 nations, more than 20% of men in this age range smoked, while 43 countries had more than 20% of females smoking. Moreover, in 12 locations, over 33% of individuals aged 15–24 were current smokers [37]. While prostate cancer primarily affects older individuals, understanding smoking trends in younger populations can help predict future cancer incidence as these individuals age. The worldwide incidence of smoking among 15-24-year-olds has decreased since 1990, although population growth has increased young smokers in several countries (Fig. 3).

### Smoking as a prostate cancer risk

Several studies have investigated the link between smoking and prostate cancer. Smokers are more likely to die from prostate cancer, which increases with obesity. A meta-analysis by Islami et al. [38] analyzed 89 cohort studies and found a positive association between smoking and prostate cancer mortality. The study concluded that current smokers had a 25% higher risk of dying from prostate cancer than nonsmokers. Furthermore, a systematic review and meta-analysis by Al-Fayez et al. [39] of 17 cohort studies revealed that current smokers had a 42% greater chance of dying from prostate cancer than nonsmokers. Multiple mechanisms are being explored to explain the association between smoking and prostate cancer. Smoking has been related to alterations in hormonal balance, oxidative stress, inflammation, and DNA damage, all of which may contribute to the development and progression of prostate carcinoma. For example, tobacco smoke contains numerous carcinogens that can lead to DNA damage, including polycyclic aromatic hydrocarbons (PAHs) and nitrosamines. Moreover, smoking has been shown to increase estrogen, androgens, and insulin-like growth factors, all associated with prostate cancer development.

### Chemicals in cigarettes

Cigarette smoking is a well-known risk factor for several types of cancer, including lung and throat cancers. Researchers have recently begun exploring the potential relationship between cigarette smoking and prostate cancer. The focus has been on the numerous chemicals in cigarette smoke, of which are known carcinogens. These chemicals include Tar, Arsenic, Cadmium, Butane, Acetic Acid, Acetone, Formaldehyde, Ammonia, Lead, and Carbon Monoxide.

Tar is a sticky substance formed when tobacco is burned. It contains several cancer-causing chemicals. Tar deposits in the lungs can also enter the bloodstream, potentially affecting other organs, including the prostate. Arsenic is a toxic element found in some pesticides that may contaminate tobacco plants. Chronic exposure to arsenic has been associated with various cancers [40]. Cadmium, a heavy metal found in cigarettes, is known to have endocrine-disrupting effects. It can mimic hormones like testosterone and has been specifically linked to prostate cancer [41]. Butane is used to keep the cigarette burning and may have toxic effects when inhaled in large quantities. These chemicals are used in various industrial applications and are also found in cigarette smoke. Formaldehyde is classified as a human carcinogen. While exposure to formaldehyde can occur in several occupational settings, it is important to clarify that it is not commonly regarded as a primary carcinogen in cigarette smoke compared to others, like tar and arsenic. Occupational exposure is a concern in certain settings, such as manufacturing plants that use formaldehyde and medical laboratories where it is used as a preservative. Due to its classification as a human carcinogen, regulations exist to control exposure levels in the workplace and consumer products. Nonetheless, understanding the risk factors and the mechanisms by which formaldehyde induces cancer is an ongoing area of research [42]. Ammonia is added to cigarettes to enhance the flavor, but it’s inhalation has been associated with several health issues. Because of the associated health risks, there is a strong public health interest in regulating cigarette additives like ammonia and educating the public on the risks associated with smoking [43]. Lead exposure is toxic, and chronic exposure may contribute to cancer development. Given the multiple pathways for potential exposure and the serious health risks associated with lead, including its possible role in cancer development, minimizing lead exposure remains a priority for public health agencies worldwide [44]. Carbon Monoxide binds to hemoglobin, reducing the oxygen-carrying capacity of the blood. While it’s not directly carcinogenic, it can contribute to overall health degradation. Although carbon monoxide itself is not classified as a carcinogen, it plays a role in reducing overall health and compromising immune responses, which can indirectly promote cancer progression. Prolonged or acute exposure to low or high quantities of it may harm health. Poor oxygenation may damage the neurological system, cardiovascular system, and cognitive and motor skills over time.

The chemicals in cigarettes lead to DNA damage, hormone disturbance (like cadmium), and inflammatory reactions in prostate cells. Some studies have found an association between cigarette smoking and a higher risk of aggressive prostate cancer, while others have reported inconsistent results. Heavy and long-term Smoking may raise prostate cancer risk, suggesting a dose-response Link. Quitting Smoking may significantly decrease exposure to these substances and may reduce prostate cancer risk. Awareness campaigns emphasizing the toxic chemicals in cigarettes and their potential Link to prostate cancer are crucial. Healthcare providers can offer targeted advice and support to those at risk, particularly heavy smokers and those with occupational exposure to these chemicals. The potential association between cigarette smoking and prostate cancer, attributed to exposure to harmful chemicals such as tar, arsenic, cadmium, butane, acetic acid, acetone, formaldehyde, ammonia, lead, and carbon monoxide, is illustrated in Table 2. These compounds cause significant harm not just to smokers but also to second-hand smokers. It emphasizes the many health risks and long-term consequences of Smoking.

**Table 2:** List of chemicals in cigarette smoke and their health effects on the human body. It provides a comprehensive overview of the 75 chemical components found in cigarette smoke, ranging from carcinogens and toxins to irritants and other constituents

S.No	Name of the Chemical	Effects on Health	References
1.	Nicotine	Highly addictive stimulant, linked to increased heart rate and blood pressure	Benowitz et al., 2009 [44]
2.	Tar	Causes lung damage and respiratory issues	Hecht et al., 1999 [45]
3.	Carbon Monoxide	Reduces oxygen-carrying capacity, leading to cardiovascular problems	Zatu et al., 2011 [46]
4.	Formaldehyde	Carcinogenic, associated with respiratory and nasopharyngeal cancers	Nielsen et al., 2010 [47]
5.	Acetone	Irritates respiratory tract, may lead to headaches and dizziness	Umeh et al., 2021 [48]
6.	Benzene	Carcinogenic, linked to leukemia and other blood disorders	Goldstein et al., 2019 [49]
7.	Ammonia	Irritates respiratory system, can exacerbate asthma	Bernasconi et al., 2022 [50]
8.	Hydrogen Cyanide	Impairs lung function, associated with respiratory failure	Guidotti, et al., 2015 [51]
9.	Lead	Toxic heavy metal, harmful to the nervous system	Fowler et al., 2023 [52]
10.	Vinyl Chloride	Carcinogenic, linked to lung cancer	Mundt et al., 2017 [53]
11.	Cadmium	Causes lung and prostate cancer, damages kidneys	Mezynska et al., 2018 [54]
12.	Polonium-210	Radioactive, associated with lung cancer	Boice et al., 2014 [55]
13.	Chromium	Carcinogenic, linked to lung cancer	Gibb et al., 2000 [56]
14.	Nitrosamines	Carcinogenic, associated with various cancers	Gushgari et al., 2018 [57]
15.	Acrolein	Irritates respiratory system, contributes to lung damage	Hikisz et al., 2023 [58]
16.	Methanol	Toxic, can cause central nervous system depression	Skrzydlewska et a l., 2003 [59]
17.	1,3-Butadiene	Carcinogenic, linked to leukemia and other cancers.	Valdez-Flores et al., 2022 [60]
18.	Toluene	Neurotoxic, can cause headaches and dizziness	Filley et al., 2004 [61]
19.	Urea	Metabolizes into harmful compounds, contributes to bladder cancer	Hofmann et al.,, 2008 [62]
20.	PAHs	Carcinogenic, linked to lung and other cancers	Moorthy et al.,, 2015 [63]
21.	Phenol	Irritates respiratory tract, can cause nausea and headache	Abd Gami et al., 2014 [64]
22.	Styrene:	Associated with respiratory issues and central nervous system effects	Banton et al., 2019 [65]
23.	Acetaldehyde	Carcinogenic, linked to liver and respiratory cancers	Salaspuro et al., 2006 [66]
24.	Propylene Glycol	Irritates respiratory system, may cause allergic reactions	Langston et al.,2021 [67]
25.	Isoprene	Irritates respiratory system, may contribute to lung damage	Doyle et al., 2004 [68]
26.	Methane	Contributes to air pollution, associated with respiratory issues	West et al., 2006 [69]
27.	Hydrogen Sulfide I	irritates eyes and respiratory tract, can cause headaches	Anenberg et al.,, 2012 [70]
28.	Benzopyrene	Carcinogenic, associated with lung and other cancers	Batterman et al., 2023 [71]
29.	Ethylbenzene	Can cause irritation of the eyes and respiratory tract	Jameson et al., 2021 [72]
30.	Hydrazine	Toxic, linked to liver and lung damage	Spencer et al., 2021 [73]
31.	Quinoline	Associated with respiratory irritation and potential toxicity	Christ et al., 1988 [74]
32.	Hexamine	Can irritate the respiratory system and cause nausea	Dreyfors et al., 1989 [75]
33.	Nitrobenzene	Toxic, associated with central nervous system effects	Wang et al., 2010 [76]
34.	Cresol	Irritates eyes, skin, and respiratory tract	Hartwig et al., 2022 [77]
35.	Cadaverine	Foul-smelling, can irritate eyes and respiratory system	Pitschmann et al., 2014 [78]
36.	Acrylonitrile	Carcinogenic, associated with lung and other cancers	Cole et al., 2008 [79]
37.	Butyric Acid	Irritates eyes, skin, and respiratory tract	Nowak et al., 2016 [80]
38.	DDT (Dichlorodiphenyltrichloroethane)	Pesticide, associated with adverse health effects	Harada et al., 2016 [81]
39.	Hydroquinone	Associated with skin irritation and potential toxicity	McGrego et al., 2007 [82]
40.	Isobutyl Acetate	Can irritate eyes, skin, and respiratory system	Heldreth et al., 2012 [83]
41.	Methacrylic Acid	Irritates eyes, skin, and respiratory tract	Borak et al., 2011 [84]
42.	Naphthalene	Carcinogenic, associated with respiratory and skin issues	Jia et al., 2010 [85]
43.	Propionaldehyde	Irritates eyes, skin, and respiratory system	Patocka et al., 2014 [86]
44.	Selenium	Heavy metal, associated with various health issues	Chen et al., 2014 [87]
45.	4-Aminobiphenyl	Carcinogenic, associated with bladder cancer	Bhattacharya et al., 2015 [88]
46.	Xylene	Can cause respiratory and central nervous system effects	Kandyala et al., 2010 [37]
47.	Phenanthrene	Carcinogenic, linked to lung and other cancers	Jameson et al., 2021 [72]
48.	Aniline	Toxic, associated with various health issues	Bomhard et al., 2005 [89]
49.	Pyridine	Can irritate the eyes, skin, and respiratory system	Tao et al., 2022 [90]
50.	2-Naphthylamine	Carcinogenic, associated with bladder cancer	Czubacka et al., 2020 [91]
51.	Dimethylnitrosamine	Carcinogenic, linked to liver cancer	Tolba et al., 2015 [92]
52.	Ethylene Oxide	Carcinogenic, associated with various cancers	Lynch et al., 2022 [93]
53.	Hexavalent Chromium	Carcinogenic, linked to lung cancer	Yatera et al., 2018 [94]
54.	Nickel	Carcinogenic, associated with lung cancer	Son et al., 2020 [95]
55.	2-Nitropropane	Toxic, associated with liver and respiratory issues	Gadagbu et al., 2020 [96]
56.	4-Methylimidazole	Carcinogenic, associated with lung and liver tumors	Brusick et al., 2020 [97]
57.	Polysorbate 80	May cause allergic reactions and skin irritation	Sözener et al., 2020 [98]
58.	Dibutyl Phthalate	Endocrine disruptor, associated with reproductive issues	Xie et al., 2019 [99]
59.	Hydroxyquinoline	May cause skin and eye irritation	Kreft et al., 2022 [100]
60.	Dioxins	Carcinogenic, associated with various health issues	Tuomisto et al., 2019 [101]
61.	Tetrachloroethylene	Carcinogenic, linked to liver and lung cancer	Klaunig et al., 2024 [102]
62.	Acetoin	Inhalation may cause respiratory irritation	Card et al., 2022 [103]
63.	Butylated Hydroxyanisole (BHA)	Possible carcinogen, associated with health concerns	Zhang et al.,, 2023 [104]
64.	Crotonaldehyde	Carcinogenic, associated with respiratory and gastrointestinal issues	IWG, 2021 [105]
65.	Diphenylamine	May cause skin and eye irritation	Chung et al., 2015 [106]
66.	1,1,1-Trichloroethane	Linked to respiratory and neurological issues	De Miranda et al., 2020 [107]
67.	Ethyl Carbamate	Carcinogenic, associated with liver and lung tumors	Dhouib et al., 2016 [108]
68.	Manganese	Neurotoxic, associated with neurological disorders	Budinger et al., 2021 [109]
69.	Naphthylamine	Carcinogenic, linked to bladder cancer	Czubacka et al., 2020 [91]
70.	Propyl Gallate	May cause skin and eye irritation	Holzer et al., 2021 [110]
71.	Pyrrole	May irritate eyes, skin, and respiratory tract	Romero et al., 2021 [111]
72.	Isophorone	Can cause skin and eye irritation	Huang et al., 2023 [112]
73.	Methyl Methacrylate	May cause respiratory and skin irritation	Pemberton et al., 2022 [113]
74.	Indole	May cause skin and eye irritation	Sun et al., 2023 [114]
75.	Vinyl Acetate	May cause respiratory and skin irritation	Albertini et al., 2013 [115]

### Risk factors other than smoking for prostate cancer

Age is one of the major risk factors for prostate cancer [116]. It is significantly associated with incidence and mortality rates, with the highest rates observed in men over 65, whereas men under 50 display distinct pathological characteristics [3]. Macneil et al. [117] investigated age-related disease disparities. Over a six-year period, researchers examined prostatectomy samples from 11,551 men and discovered unknown age discrepancies. Younger males were more likely to have low-grade or early-stage cancers. However, it is important to note that age is more than just the number of Years; it also includes the biological Changes that come with it. A separate, large study examined the records of 391,068 men after radical prostatectomy and found that younger men had worse oncological results. Such data led researchers to suggest 50 as a benchmark age for prostate cancer patient categorization [118]. In addition to age, genetics play a major role in prostate cancer risk [119]. Ethnicity is another crucial element in prostate cancer prevention. Men of African ancestry have a much greater incidence, severity, and death rate from prostate cancer [120]. In the United States, significant disparities occur across ethnic and racial groupings [121]. Black males of African descent had the greatest incidence, over three times that of other ethnic groupings [122]. Alarmingly, their mortality rate is two to four times higher than that of White men [123]. These disparities may stem largely from differences in diagnostic practices and healthcare access. Within the United States, African-American males not only have greater incidence rates, but they also have more aggressive types of the cancer than white men. Beyond genetics and ethnicity, lifestyle and environmental factors also shape risk. Obesity (BMI ≥ 30) raises the risk of aggressive prostate cancer, whereas regular exercise helps mitigate it [124]. Omega-3-rich seafood may also protect against the illness [125]. However, calcium consumption beyond the RDA may raise risk [126]. Higher vitamin D levels may protect against prostate cancer, although some studies find no link [127].Langlais et al. [128] investigated the complex relationship between post-diagnostic coffee and tea intake and prostate cancer progression. The median study time was nine years, with 1,557 male participants. To obtain data, prostate cancer patients were given a complete eating frequency questionnaire 28 months following their diagnosis. This questionnaire asked about their coffee and tea use, as well as their smoking status (current, former, or nonsmoker). Over the course of the extended study, the researchers discovered 167 cases of prostate cancer progression. They observed a strong association between coffee intake and prostate cancer progression, strongest among current smokers—suggesting a possible interaction between smoking and coffee. Tea intake did not seem to be linked to prostate cancer, regardless of smoking status. Although these results provide important new information on possible dietary elements that can affect the development of prostate cancer, particularly in the context of concurrent coffee use and smoking, it is important to emphasize that correlations can be made only from observational research. Moreover, future studies should further investigate potential confounders underlying these correlations.

In addition, emerging meta-analytic data highlights the importance of occupational exposures in prostate cancer risk A comprehensive meta-analysis by Krstev and Knutsson (2019) reviewed 168 studies and found significantly increased risks associated with occupational exposure to pesticides (metaRR = 1.15, 95% CI: 1.01–1.32), organochlorine pesticides (metaRR = 1.08, 95% CI: 1.03–1.14), chromium (metaRR = 1.19, 95% CI: 1.07–1.34), and shift work (metaRR = 1.25, 95% CI: 1.05–1.49) [129]. In contrast, general population studies on smoking report weaker associations with prostate cancer incidence, typically with relative risks below, though smoking is more clearly linked to disease progression and mortality [133;134]. These findings suggest occupational exposures may confer a slightly higher prostate cancer risk compared to general lifestyle factors such as smoking, emphasizing the need for targeted preventive strategies in occupational settings. Certain foods may increase or reduce risk. However, lycopene-rich diets may lower prostate cancer risk [179].

**Fig. 4:**
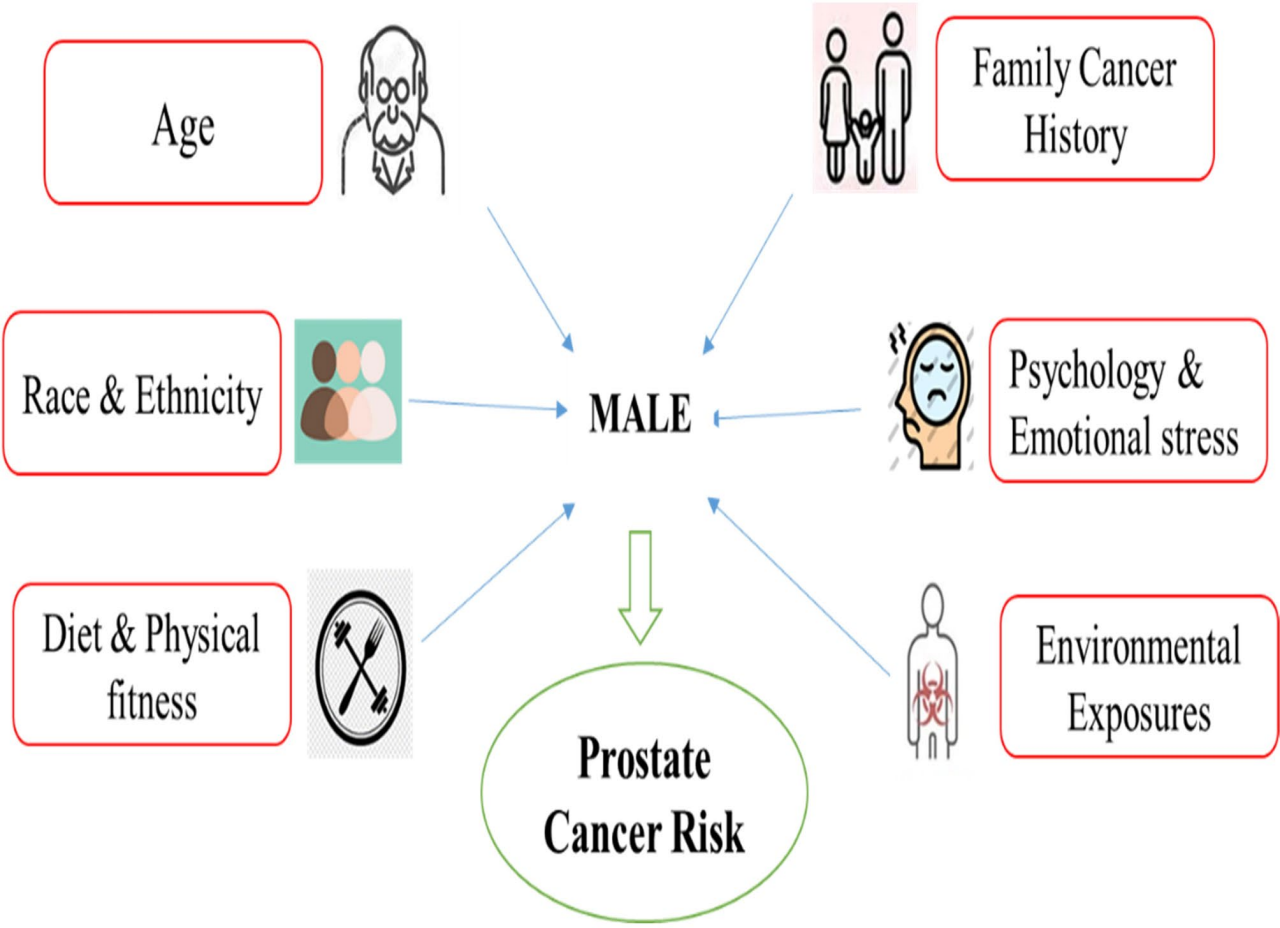
Illustration of major prostate cancer risk factors in males. The diagram includes genetics (e.g., age, family history), demographic (race and ethnicity), lifestyle (diet, physical fitness, smoking), psychological (emotional stress), and environmental exposures as potential contributors to prostate cancer risk

Although definitive preventive actions for prostate cancer are still challenging, a low-fat diet high in fruits and vegetables, as well as consistent exercise, show great risk-reducing power. Psychological and emotional stress, often unnoticed, may indirectly affect prostate cancer growth and progression. Chronic stress, though less well-known than smoking or hereditary risk, can impair hormonal and immunological control, promoting cancer growth. Stress can cause carcinogenesis through elevated cortisol, inflammation, and oxidative stress. The fight against prostate cancer is essentially complex, modifiable and non-modifiable risk factors linked to prostate cancer all playing a part (Figure. 4). Ongoing research is essential for broadening our understanding and enhancing prevention, diagnosis, and treatment methods.

### Cadmium and androgen receptor

Scientists and healthcare professionals are concerned about the potential link between cadmium exposure and prostate cancer, as cadmium is a heavy metal present in cigarette smoke, certain foods, and industrial settings. Workers in industries like batteries, pigments, and coatings may be exposed to cadmium, a significant source of cadmium exposure. Among the numerous harmful compounds in cigarette smoke, cadmium stands out for its particular link to prostate health. Certain foods like shellfish and organ meats also contain cadmium, though the association between dietary cadmium and prostate cancer is less well-established [130]. The relationship between cadmium and prostate cancer is not straightforward [131]. Cadmium’s carcinogenic effects on the prostate are not caused by direct mutations. Cadmium interacts with the androgen receptor, a key player in prostate cancer development, and research suggests it can enhance the response of the androgen receptor in human prostate cancer cell lines by mimicking hormonal activity similar to testosterone [132]. This heightened activity could increase the risk of developing prostate cancer [133]. Animal studies suggest a link between cadmium exposure and prostate cancer, especially in occupational settings, but inconsistent results emphasize the complexity of cancer development and the need for prevention and awareness. Understanding the cadmium–androgen receptor interaction may reveal future therapeutic targets.

### Hormonal changes and prostate cancer

The male reproductive system functions effectively when androgens (such as testosterone) and oestrogens are in balance. Cigarette smoking disrupts this balance, which may lead to a variety of health diseases, including prostate cancer [134–136]. Smoking delivers toxic chemicals into the body and may disrupt the endocrine system, which controls hormone synthesis and regulation. In smokers, elevated levels of bioavailable testosterone may directly impact prostate health. Testosterone, especially its potent derivative, Dihydrotestosterone (DHT), is essential for optimal prostate gland function and development [137]. Elevated amounts of these hormones may promote fast cell proliferation, possibly leading to malignancy. Testosterone and androstenedione, both androgens, are known to promote cell proliferation. Research showing positive correlations between cigarette consumption and elevated serum levels of these hormones indicates a heightened risk factor for disorders linked to excessive cell growth, such as cancer. Estradiol, a form of estrogen, typically counterbalances male testosterone. In smokers, reduced estradiol levels may limit its regulatory functions [138]. Estrogens can inhibit the release of gonadotropins from the hypothalamus and pituitary gland, reducing androgen production in the testicles. This balancing act can be protective against excessive prostate growth and other testosterone-driven processes. Smoking can shift the balance in favour of prostate cancer growth by lowering oestradiol levels. When these hormonal alterations are combined, it is clear that smoking might create an internal environment that promotes prostate cancer progression with increased testosterone and decreased estrogen. The prostate may experience increased cellular proliferation and decreased control, setting the framework for potentially cancerous alterations. Thus, understanding smoking-induced hormonal shifts underscores the importance of cessation, especially for those at risk of prostate disease [139].

### Epidemiological insights and challenges

The relationship between smoking and prostate cancer risk remains complex and multifaceted, reflecting the interplay of genetic, environmental, and lifestyle factors. Mechanistic studies provide strong biological plausibility for smoking as a contributor to prostate carcinogenesis. Tobacco smoke contains numerous carcinogens capable of inducing DNA damage, oxidative stress, and inflammatory responses, all of which can promote tumor initiation and progression. Experimental models have demonstrated how smoking-related genotoxic stress may interact with inherited genetic susceptibilities, such as polymorphisms in DNA repair genes, to influence prostate cancer development. These mechanistic insights underscore the potential for smoking to exacerbate disease aggressiveness and impact clinical outcomes [143–145].

In contrast, epidemiological evidence linking smoking to prostate cancer incidence is less consistent. Large-scale cohort and case-control studies have often reported weak or null associations between smoking and the risk of developing prostate cancer [146;147]. This discrepancy is partly attributable to the influence of prostate-specific antigen (PSA) screening, which has led to widespread overdiagnosis of indolent tumors, thereby obscuring true etiological relationships. Recent meta-analyses underscore these inconsistent findings, with some cohorts even reporting a lower incidence among smokers—possibly reflecting detection bias, residual confounding, or lower screening rates in this group. Moreover, confounding factors such as age, ethnicity, and smoking intensity further complicate the interpretation of epidemiological data [134;148;149].

**Fig. 5:**
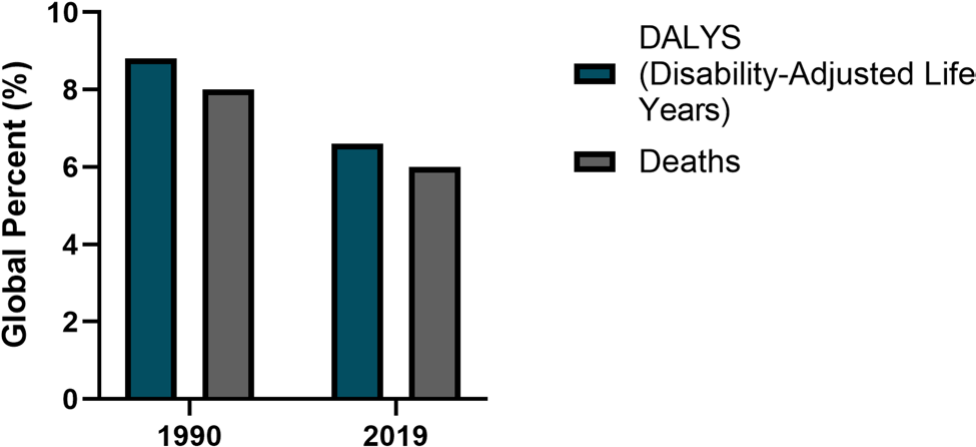
The percentage proportion of prostate cancer deaths and disability-adjusted life years (DALYs) attributable to smoking globally between 1990 and 2019 contributed to approximately 8.3%, 6%, and 8.8%, 6.6%, respectively

Importantly, recent meta-analyses and systematic reviews have shifted the focus from incidence to mortality outcomes, revealing a more robust association between smoking and prostate cancer-specific mortality. smokers have a 24–42% higher risk of death from prostate cancer compared to non-smokers, with a clear dose–response relationship between the number of cigarettes smoked and mortality risk Current smokers face a 24–42% greater risk of prostate cancer mortality than non-smokers, with a clear dose–response effect based on cigarette consumption. For instance, smokers have been shown to experience significantly higher risks of fatal prostate cancer and poorer survival compared to non-smokers (133, 134, 150). This distinction is vital, as mortality more accurately reflects clinically significant disease burden and public health impact [Fig. 5]. These findings emphasize the necessity of prioritizing mortality outcomes in both research and public health interactions, rather than highlighting the impact of smoking on incidence. Therefore, future research should prioritize longitudinal studies with detailed exposure assessment and molecular characterization to elucidate the pathways linking smoking to aggressive prostate cancer phenotypes. Integrating genomic data with environmental exposures will enhance risk stratification and inform targeted prevention and treatment strategies. Meanwhile, public health messaging should accurately convey that smoking cessation offers tangible benefits in reducing prostate cancer mortality, even if its effect on incidence remains modest.

### Smoking cessation and prostate cancer risk

Smoking cessation is strongly associated with both a reduced risk of developing prostate cancer and improved outcomes in men already diagnosed with the disease. Benefits include risk reduction, delayed disease progression, Hormonal modulation, and enhanced therapeutic response. Multiple studies have shown a clear association between smoking and elevated prostate cancer risk. Long-term cessation typically for 10 years or more can reduce the risk to a level comparable with that of men who have never smoked [5]. A meta-analysis of 13 studies reported that current smokers have a 28% higher risk of developing prostate cancer compared to non-smokers, whereas former smokers showed only a 10% increased risk, indicating substantial benefit from cessation [146]. Continuing to smoke after diagnosis is linked to more aggressive disease, higher recurrence, and increased mortality [10]. Smoking cessation may also enhance treatment efficacy, particularly in patients undergoing radiation therapy. Patients who smoke during radiation treatment show poorer response rates and a higher risk of adverse effects [63]. Therefore, cessation prior to therapy is recommended to optimize response and minimize complications.

Yang et al. [147] reviewed the link between smoking cessation and prostate cancer, concluding that quitting is associated with a reduced risk of disease development. This protective effect may be mediated by changes in sex hormone levels. Nolazco et al. [148] further examined how smoking cessation affects prostate cancer incidence and mortality. Their findings supported that quitting smoking reduces prostate cancer risk, potentially due to biological mechanisms. These mechanisms include hormonal regulation, reduced DNA damage, and improved immune function. Brookman-May et al. [149] also explored the impact of smoking cessation on prostate cancer risk. Their review highlighted recent evidence suggesting that cessation may lower risk through mechanisms involving hormonal changes, DNA repair, and reduced inflammation. Overall, available evidence supports that smoking cessation is associated with a reduced Likelihood of developing prostate cancer. A meta-analysis of 13 studies reported that current smokers have a 28% higher risk of developing prostate cancer compared to non-smokers. In contrast, former smokers had only a 10% increased risk, indicating a significant reduction following cessation [150].

Quitting smoking after diagnosis can also improve survival. Liu et al. [151] reported that men who stopped smoking post-diagnosis had lower prostate cancer–specific mortality than those who continued. Although mechanisms remain uncertain, smoking-related inflammation, oxidative stress, and DNA damage are thought to worsen outcomes [152].

Several interconnected factors—including sex hormone levels, oxidative stress, DNA damage, immune function, and inflammation—contribute to prostate cancer susceptibility. This underscores the need for comprehensive evaluations to better understand the relationship between smoking cessation and prostate cancer risk. Quitting smoking offers multiple benefits, such as reducing the likelihood of developing prostate cancer, improving treatment outcomes, and lowering the risk of disease progression. Moreover, hormonal changes induced by smoking cessation may enhance responsiveness to treatment. Thus, smoking cessation is beneficial for individuals diagnosed with prostate cancer by reducing disease risk and to optimize prognosis [158–160].

### Public health implications

Future research should also look at the effect of second-hand smoke exposure on prostate cancer risk, as well as the possible influence of e-cigarettes and other types of tobacco usage. From a public health standpoint, understanding the association between smoking and prostate cancer may help to focus preventive measures. Public health campaigns can highlight prostate-specific risks associated with smoking, encouraging men to quit or never start in the first place. Prostate cancer patients may benefit from smoking cessation programs and support services. Healthcare providers can also help patients quit smoking. Integrating smoking cessation support into routine healthcare, particularly for men at risk or diagnosed with prostate cancer, can lower smoking rates and enhance patient outcomes. Future research and public health initiatives should emphasise smoking cessation as a key tool to reduce prostate cancer risk and improve prognosis. Since smoking may cause prostate cancer, specific smoking cessation strategies should be used. These initiatives should address the needs and challenges of smokers at risk of or with prostate cancer. Smoking cessation treatments include behavioural counselling, prescription drugs, nicotine replacement therapy, and medical or support group help [156]. Encouragement and assistance in quitting smoking can dramatically lower prostate cancer risk and enhance health. Healthcare and public health groups are essential to quitting smoking. Public health campaigns, educational materials, and healthcare practitioner training can increase awareness about smoking and prostate cancer. Smoke-free policies and access to cessation tools can support individuals trying to quit. Smoking cessation is crucial due to prostate cancer and public health dangers. Understanding, helping, and lowering smoking and related health hazards require ongoing public health studies and actions.

## Conclusions

We presented a detailed examination of the effect of smoking on prostate cancer. Given the probable link, specific smoking cessation techniques should be employed, tailored to the needs of those at risk or diagnosed with prostate cancer. The benefits of smoking cessation are significant, with evidence linking quitting to decreased disease progression and higher overall survival among men with prostate cancer [157]. Major findings from substantial studies (Supplementary Table 1) indicate that smoking increases prostate cancer risk. Importantly, smokers have a worse prognosis than nonsmokers [158], with higher rates of recurrence, progression, mortality, and increased treatment complications such as surgery and radiation difficulties. Smoking may contribute to more aggressive prostate cancer forms, although the mechanisms remain under research. Exposure to second-hand smoke may also increase the risk of prostate cancer, however this link is less defintive. The link between smoking and prostate cancer is complex, including both lifestyle decisions and genetic predisposition. Some studies indicate an elevated risk, particularly for aggressive cases, whereas others show equivocal relationships [152;153;164].

Genetic polymorphisms affecting tobacco carcinogen metabolism, DNA repair, cell cycle regulation, and androgen receptor signalling may modify smoking-related prostate cancer risk [165, 166]. Identifying these factors could enable prevention [166;167].

Prostate cancer predominantly affects men over 65 due to accumulated genetic mutations, hormonal changes, and other age-related biological processes. However, younger men also reported developing the disease. According to the study in Cytecare Hospital, Bangalore (2022), prostate cancer is a growing concern among younger urban men aged 35–44 and 55–64, with cases reported in men as young as 15. Emerging risk factors contributing to rising cases include obesity, sedentary lifestyle, HPV infection, and environmental pollutants. Advances in imaging and biomarker discovery have enabled early detection in younger populations, expanding treatment options [163].

Molecular profiling is revolutionizing the clinical management of prostate cancer by distinguishing aggressive tumors from indolent forms, helping to reduce concerns about overdiagnosis and overtreatment. This approach highlights the need for refined prognostic markers to avoid unnecessary interventions. Genetic studies show that polymorphisms in DNA repair genes (e.g., GSTP1) and inflammation-related genes (e.g., IL6) influence risk linked to smoking and environmental exposures, emphasizing the importance of integrative research that combines genetics with lifestyle and environmental factors [169, 173, 179].

Future research through large-scale, multiethnic longitudinal studies and clinical trials is crucial to establish a cause-and-effect relationship, provide conclusive insights into genetic factors, and fully understand the impact of smoking on prostate cancer biology, potentially revealing therapeutic targets and fully understand the effects of smoking on prostate cancer biology, potentially revealing therapeutic targets. The integration of Genetic, molecular, and epidemiological insights will enable personalized prevention and treatment strategies. Continued emphasis on smoking cessation programs and multidisciplinary collaboration is essential to improve patient outcomes and reduce the global burden of prostate.

## Supplementary Information

Supplementary Material 1. Supplementary Table 1 Summary of selected key reference quotes in this article.

## Data Availability

No datasets were generated or analysed during the current study.
