# Supplementary material for: Smoking and the risk of prostate cancer: a review of risk and disease progression
Source: Genes Environ. 2025 Oct 9;47:17. doi: 10.1186/s41021-025-00338-8 (PMC12512299; doi:10.1186/s41021-025-00338-8)
Supplement: Supplementary file 1 — Supplementary Material 1. Supplementary Table 1 Summary of selected key reference quotes in this article. [file 41021_2025_338_MOESM1_ESM.docx]

**Supplementary Table 1:** Summary of selected key reference quotes in this article.

| **Ref. NO** | **References** | **Study goals** | **Methodology** | **Results** | **Conclusions** |
| --- | --- | --- | --- | --- | --- |
| 2 | Al-Fayez et al., (2023) | This study examines this association using a systematic review and meta-analysis of updated evidence. | The study analyzed the relative risk and 95% confidence intervals of prostate cancer incidence among smokers and non-smokers in prospective cohort studies using PubMed and Scopus databases. | A systematic review of 17 studies found cigarette smoking inversely associated with prostate cancer incidence,while current smokers had a 42% higher risk of death from prostate cancer (95% CI: 1.20–1.68 | Smoking has an inverse association with prostate cancer incidence, but smokers have an increased risk of death due to low screening uptake, misclassification bias, and selection bias. |
| 3 | Rohrmann et al., (2013) | This prospective study aims to find smoking and the risk of prostate cancer in the European population | Lifestyle information in European men from 1992-2000 revealed 4623 incident cases of prostate cancer, with smoking status, intensity, and duration correlated with risk. | Current smokers have a reduced risk of prostate cancer (RR=0.90, 95% CI: 0.83–0.97), while heavy smokers (25+ cigarettes per day)  and long-time smokers (40+ years) have a higher risk of prostate cancer death (RR=1.81, 95% CI: 1.11–2.93; RR=1.38, 95% CI: 1.01–1.87, respectively). | The study confirms earlier findings that heavy smokers are more likely to develop prostate cancer. |
| 10 | Gansler et al., (2018) | The study investigates the specific mortality rates associated with smoking and prostate cancer diagnosis in a large prospective cohort. | The study analyzed non-metastatic prostate cancer cases from 1992-1993, focusing on pre-diagnosis and post-diagnosis smoking, with vital status follow-up through 2014. | Prostate cancer deaths are linked to smoking before [HR = 1.50; 95% confidence interval (CI), 1.06–2.13] and after diagnosis (HR = 1.71; 95% CI, 1.09–2.67), with smokers who quit smoking less than 20 years before diagnosis at higher risk (HR = 1.29; 95% CI, 1.04–1.6) | The study shows that smoking before and after a prostate cancer diagnosis is linked to higher PCSM, even after considering stage and Gleason score. |
| 20 | Gupta et al., (2020) | A pilot study using whole exome sequencing of benign/PCa patients in India identified specific mutations. | Whole exome sequencing | Numerous mutations in DNA repair genes, including helicases, TP53, and BRCA, were observed, along with a potentially damaging rare variant in the TNNI3 gene. | The pilot study aims to provide insight into the prognosis and recurrence of PCa in the Indian phenotype. |
| 23 | Kenfield et al., (2011) | The study investigates the correlation between cigarette smoking and cessation, overall, prostate cancer-specific, and cardiovascular disease mortality and biochemical recurrence in prostate cancer men. | The Health Professionals Follow-Up Study, a prospective observational study, analyzed 5366 men diagnosed with prostate cancer between 1986 and 2006. | The study found that current smokers have an increased risk of prostate cancer mortality, biochemical recurrence, total mortality, and CVD mortality compared to non-smokers. Those with 40 or more pack-years of smoking had a higher risk of prostate cancer mortality. Quitting smoking for ten or more years or less than 20 pack-years had similar risks. | Smoking at the time of prostate cancer diagnosis increases overall CVD mortality, as well as prostate cancer-specific mortality and recurrence. Quitting smoking for at least ten years has similar risks to never smoking. Studies suggest a 30% increase in fatal prostate cancer risk. |
| 34 | Culp et al., (2020) | The study aims to evaluate the current global incidence and mortality rates of prostate cancer using existing data. | The study presents 2018 prostate cancer incidence and mortality rates based on the GLOBOCAN database and examines trends using data from 44 countries and 76 countries. | Prostate cancer incidence and mortality rates vary across countries, with the highest rates in Australia/New Zealand, Northern America, Western Europe, and the Caribbean, and lowest rates in South-Central Asia, Northern Africa, and Asia. | Prostate cancer incidence and mortality rates have been declining or stabilizing in many countries, particularly in high-income nations, due to decreased prostate-specific antigen testing and improved treatment. |
| 122 | Zi et al., (2021) | The study aims to explore the global and regional burden of genitourinary cancers and the risk factors associated with them over the past 30 years. | The study analyzed data on kidney, bladder, and prostate cancers from 1990 to 2019, examining changes in incidence, mortality, and risk factors and their associations with socio-demographic characteristics. | Global incidence of kidney, bladder, and prostate cancers increased by 154.78%, 123.34%, and 169.11% in 2019 compared to 1990—lower ASMR and ASDR for bladder and prostate cancers but higher mortality rates. | Kidney, bladder, and prostate cancers pose global public health challenges, requiring proactive intervention strategies at administrative and academic levels to adapt to regional and socioeconomic trends. |
| 147 | Huncharek et al., (2010) | The study assessed the correlation between smoking and prostate adenocarcinoma | A meta-analysis of 24 cohort studies involving 21 579 prostate cancer participants was conducted, assessing the robustness of effect measures and evaluating statistical heterogeneity. | Current smokers have no increased risk of prostate cancer (RR = 1.04; 95% CI = 0.87, 1.24), but those who smoke significantly more have a higher risk of fatal prostate cancer (RR = 1.14; 95% CI = 1.06, 1.19). | Observational studies show a link between smoking and prostate cancer incidence and mortality but often lack clear exposure categories, suggesting an underestimation of risk in pooled data. |
| 149 | Yang et al., (2023) | The systematic review and meta-analysis aimed to evaluate the correlation between cigarette smoking and prostate cancer risk. | A systematic search was conducted on PubMed, Embase, Cochrane Library, and Web of Science to examine the association between cigarette smoking habits and prostate cancer risk. | A meta-analysis of 7296 publications found that current smokers have a significantly reduced risk of prostate cancer  (RR, 0.74; 95% CI, 0.68–0.80; P < 0.001),  especially in prostate-specific antigen screening eras, and a lower risk of PCa compared to former smokers  (RR, 0.70; 95% CI, 0.65–0.75; P < 0.001) | The study suggests that smokers' lower prostate cancer risk may be due to poor cancer screening adherence and smoking-related diseases, suggesting measures to encourage early screening and quit smoking. |
| 154 | Brookman-May et al., (2019) | The study assesses the correlation between smoking, sexual activity, and sports and exercise on PCa risk, treatment outcomes, progression, and cancer-specific mortality. | A systematic review of studies published between 2007 and 2017 was conducted using MEDLINE, Cochrane Central Register of Controlled Trials, and Web of Science databases. | Smoking is linked to aggressive tumor features and worse cancer outcomes, while sexual activity and physical activity may prevent PCa and tumor progression.  The systematic review explores the impact of smoking, sexual activity, and sports on prostate cancer risk and treatment outcomes, suggesting that an active lifestyle is recommended. | Quitting smoking at PCa diagnosis can improve prognosis, while exercise positively affects tumor development and treatment. Further research is needed to decide the relationship between sexual activity and PCa risk. |
| 155 | Chan et al., (2018) | This study examines potential associations between serum testosterone, dihydrotestosterone, and estradiol with overall cancer risk, prostate, colorectal, and lung cancer risk, excluding skin cancer. | The study examined cancer risk in 1,574 men aged 25-84 years. Results showed that androgens in the lowest quartile increased overall cancer risk, with T in the lowest quartile increasing prostate cancer risk. | The study found that androgens in the lowest quartile increased overall cancer risk, particularly prostate cancer. However, there were no significant associations with colorectal or lung cancer risk. | Further confirmation is needed on the role of T as a biomarker for poor health in men with cancer or prostate cancer and the association between high LH and future lung cancer. |
| 160 | Tang et al., (2017) | The study aims to explore the correlation between smoking and various prostate cancer pathological subtypes in Chinese men. | A study involving 1795 prostate biopsy patients from 2013 to 2016 used logistic regression to assess the link between cigarette smoking and prostate cancer incidence. | A study found that current smokers have a higher risk of high-grade prostate cancer (HGPCa) and intraductal carcinoma (OR = 1.89, 95%CI: 1.44-2.48) of the prostate (IDC-P) (OR = 2.29, 95%CI: 1.14-4.59) compared to never smokers. | The Chinese biopsy cohort found that current smoking among men is linked to highly malignant PCa incidence, including HGPCa and IDC-P, and may also be associated with smoking duration. |
| 172 | John et al., (2021) | The study estimates the economic impact of smoking and smokeless tobacco use on individuals aged 35 and above. | The economic burden of diseases and deaths due to tobacco use in India is estimated using data from healthcare expenditures, prevalence, and mortality risks. | In India, tobacco use caused $27.5 billion in economic costs in 2017-2018, with men bearing 91% of the total costs, smoking contributing 74%, and SLT use contributing 26%. | Tobacco use costs India 1.04% of GDP, affecting health care and the economy. Scaling up tobacco control efforts is necessary to reduce stress on public health and the economy. |
| 173 | Amor et al., (2023) | The study investigated transcript levels of PGAM5, PTPRN2, and TYRO3 in heavy smokers compared to non-smokers and their association with fundamental sperm parameters. | This study involved 118 sperm samples, 63 heavy smokers and 55 non-smokers, and performed semen analysis according to WHO guidelines. Total RNA extraction and RT-PCR quantification were used to study gene transcript levels. | Smokers in G1 showed a significant decrease in standard semen parameters, differential expression of genes like PGAM5 and PTPRN2, and negative correlation with motility, sperm count, vitality, and membrane integrity. | Smoking can affect male fertility by altering DNA methylation patterns in genes related to fertility and sperm quality, such as PGAM5, PTPRN2, and TYRO3. |
| 174 | Cao et al., (2013) | This meta-analysis aimed to evaluate the link between smoking and the risk of erectile dysfunction (ED) by synthesizing evidence from numerous studies. | In 2013, researchers analyzed studies on smoking and erectile dysfunction (ED), assessed study quality using the Newcastle-Ottawa scale and conducted random-effects meta-analyses to combine results. | Four prospective cohort studies and four case-control studies involving 28,586 participants found consistent results in erectile dysfunction (ED) rates for current smokers and former smokers, with no evidence of publication bias. | Epidemiological studies show that smoking, particularly current smoking, may significantly increase the risk of erectile dysfunction (ED). |
| 175 | Ho et al., (2014) | The REDUCE study, which followed biopsy-negative men for 2 and 4 years, found a link between smoking and prostate cancer mortality, despite inconsistent evidence. | A logistic regression study was conducted to examine the correlation between smoking and cancer in the first on-study biopsy in REDUCE. | A study of 6,240 men found that smokers were less likely to receive a second on-study biopsy, and those with a BMI ≤ 25 kg/m2 had an increased risk of prostate cancer. | In REDUCE, men with elevated PSA and negative pre-study biopsy showed smoking was unrelated to overall prostate cancer diagnosis but increased the risk of high-grade prostate cancer. |
| 176 | Foerster et al., (2018) | The study aims to analyze the correlation between smoking status, biochemical recurrence, metastasis, and cancer-specific mortality in patients with localized prostate cancer undergoing radical prostatectomy or radiotherapy. | A systematic review and meta-analysis of original articles published between 2000 and 2017 examined Cox proportional hazards regression and logistic regression analyses, including multivariable hazard ratios and risk-of-bias assessments. | A study of 5157 reports found that current smokers have a higher risk of biochemical recurrence, metastasis, and cancer-specific mortality compared to former smokers, with a median follow-up of 72 months. | Smokers currently receiving primary curative treatment for localized prostate cancer are at a higher risk of biochemical recurrence, metastasis, and cancer-specific mortality. |
| 177 | Dwivedi et al., (2012) | The study investigates pro-inflammatory levels in prostate carcinoma patients by examining IL-18 expression levels in tobacco-exposed individuals. | A study recruited 578 prostate cancer patients and 294 controls, analyzing their IL-18 levels using ELISA. | IL-18 levels significantly differed between cancer patients and controls, with higher levels observed in smokers and chewers (P < 0.05) and higher stages of cancer. | This study reveals that tobacco exposure, a risk factor for various cancers, significantly enhances inflammation in prostate carcinoma patients, particularly in stratified groups. |
| 178 | Murphy et al., (2013) | The study investigates the impact of tobacco use on the likelihood of high-grade prostate cancer in a predominantly African-American male population. | The study surveyed 1,085 men aged 40+ in two US cities from 2001 to 2012, examining smoking and PCa status in men with PCa and healthy controls. | The study found that African American men are more likely to smoke and quit than European American men, with increased odds of PCa diagnosis and high-grade cancer. | Ethnic differences in smoking behavior were discovered, with heavy smoking linked to higher PCa odds and higher Gleason grades in African-American men. |
| 179 | Roddam et al., (2008) | The study aims to evaluate the correlation between the levels of IGFs and IGFBPs and the subsequent risk of prostate cancer. | The studies were sourced from sources such as PubMed, Web of Science, and Cancer Lit. | The study found that higher serum IGF-I concentrations increased prostate cancer risk. However, IGFBP-III concentration was associated with prostate cancer risk, but this was secondary to IGF-I levels. No significant heterogeneity was found. | Elevated levels of IGF-I in the blood are linked to a moderately elevated risk of prostate cancer. |
| 180 | Jain et al., (2014) | The review article focuses on the epidemiology of prostate cancer in the Indian subcontinent, examining its incidence, survival, and mortality rates. | The article presents the incidence rates, mortality, and trends over time for prostate cancer as the data collected from national population-based cancer registries. | The PBCRs at Bangalore (Annual Percentage Change: 3.4%), Chennai (4.2%), Delhi (3.3%), Mumbai (0.9%), and Kamrup Urban District (11.6%) recorded a statistically significant increasing trend in incidence rates over time. | India's cancer incidence rates are increasing rapidly across all PBRCs, with projections predicting a doubled number of cases by 2020. |
| 181 | Shiota et al., (2019) | The study aimed to understand the biological impact of cigarette smoking on AR signaling and its clinical impact on oncological outcomes. | The study evaluated gene expression, cellular sensitivities, and prognosis in prostate cancer cells exposed to tobacco smoke condensate, enzalutamide, and docetaxel, comparing current smokers and nonsmokers. | TSC exposure increases AR variant and prostate-specific antigen expression, suppressed by Akt inhibitor LY294002 and antioxidant N-acetylcysteine. Cigarette smoking negatively impacts progression-free and cancer-specific survival in prostate cancer patients. | Cigarette smoking negatively impacts oncological outcomes in prostate cancer patients treated with ARAT agents, suggesting a recommendation to avoid smoking in advanced cases. |
| 182 | Plaskon et al., (2003) | The study aimed to evaluate the correlation between age and health in middle-aged men through a population-based case-control approach. | The study involved 753 Men diagnosed with prostate cancer from 1993-96 and 703 controls without cancer and used logistic regression to assess the prostate cancer-cigarette smoking relationship. | Smoking increases prostate cancer risk, with a dose-response relationship between pack-years smoked and risk (trend P = 0.03). The OR = 1.6 (95% CI 1.1–2.2). Men over 40 pack-years have a stronger association (OR = 2.0, 95% CI 1.3–3.1), and cessation results in a decline in risk (P = 0.02). | Smoking increases prostate cancer risk, with a dose-response relationship. Smoking cessation reduces risks, suggesting prostate cancer should be added to cancers with smoking-related risk factors. |
| 183 | Tsai et al., (2018) | The study investigates the impact of smoking on adipose tissue, focusing on the coordinated changes in DNA methylation and gene expression, which could potentially impact metabolic health. | The study examined smoking-associated DNA methylation and gene expression variations in adipose tissue biopsies from 542 healthy female twins, examining replication, tissue specificity, and longitudinal stability. | The study identified 42 smoking-methylation and 42 smoking-expression signals, with five genes hypo-methylated and upregulated in current smokers. Smoking impacts DNA methylation, affecting metabolic disease risk traits. | The study reveals the first comprehensive analysis of DNA methylation and gene expression markers of smoking in adipose tissue, providing insights into its widespread health consequences. |

**Supplementary Information**

**Supplementary Table 1:** Summary of selected key reference quotes in this article.
